# Differential Profiles of Gut Microbiota-Derived Metabolites of Bile Acids and Propionate as Potential Predictors of Depressive Disorder in Women with Morbid Obesity at High Risk of Metabolic Dysfunction-Associated Steatotic Liver Disease—A Pilot Study

**DOI:** 10.3390/cimb47050353

**Published:** 2025-05-12

**Authors:** Joanna Michalina Jurek, Belen Xifré, Elena Cristina Rusu, Helena Clavero-Mestres, Razieh Mahmoudian, Carmen Aguilar, David Riesco, Javier Ugarte Chicote, Salomé Martinez, Marga Vives, Fàtima Sabench, Teresa Auguet

**Affiliations:** 1 Grup de Recerca GEMMAIR (AGAUR)—Medicina Aplicada (URV), Departament de Medicina i Cirurgia, Universitat Rovira i Virgili (URV), Institut d’Investigació Sanitària Pere Virgili (IISPV), Mallafré Guasch, 4, 43007 Tarragona, Spain; joanna.michalina.jurek@gmail.com (J.M.J.); elena.cristina.rusu@gmail.com (E.C.R.); helena.clavero@urv.cat (H.C.-M.);; 2 Servei Medicina Interna, Hospital del Vendrell, Ctra. Barcelona, s/n, El Vendrell, 43700 Tarragona, Spain; 3 Servei Medicina Interna, Hospital Universitari de Tarragona Joan XXIII, Mallafré Guasch, 4, 43007 Tarragona, Spain; 4 Servei Anatomia Patològica, Hospital Universitari de Tarragona Joan XXIII, Mallafré Guasch, 4, 43007 Tarragona, Spain; 5 Servei de Cirurgia, Hospital Sant Joan de Reus, Departament de Medicina i Cirurgia, Universitat Rovira i Virgili (URV), Institut d’Investigació Sanitària Pere Virgili (IISPV), Avinguda Doctor Josep Laporte, 2, 43204 Reus, Spain

**Keywords:** depression, metabolic dysfunction-associated steatotic liver disease, bile acids, microbial metabolites, obesity

## Abstract

Metabolic dysfunction-associated steatotic liver disease (MASLD) is a liver condition linked to cardiometabolic diseases and mental health issues, with studies highlighting disruptions in gut microbiota activity, including bile acid (BA) metabolism. Therefore, the main aim of this exploratory analysis was to assess microbiota-derived metabolites, specifically BAs and short-chain fatty acids (SCFAs), as potential biomarkers of depressive disorder (DD) in women with morbid obesity at MASLD risk. In this pilot study, 33 females with morbid obesity who were scheduled for bariatric surgery were evaluated. Medical and clinical data were collected, and microbial metabolites from pre-surgery blood samples were analyzed. Patients were stratified according to the presence of DD. Analysis with Spearman’s rank test was used to assess correlations and logistic regression models were built to evaluate biomarkers as predictors of DD risk using both receiver operating characteristic (ROC) and precision–recall curves. In this cohort, 30.3% of females were reported to have DD, in addition to significantly elevated levels of certain BAs and SCFAs, including glycodeoxycholic acid (GDCA) and propionate, which were also correlated with some metabolic biomarkers. However, there were no differences in the incidence of MASLD or metabolic syndrome between patients with DD or without. In conclusion, microbiota-derived metabolites such as GDCA and propionate may influence DD risk in females with morbid obesity; however, their potential use as predictive biomarkers should be further investigated to confirm their role in psycho-metabolic conditions.

## 1. Introduction

Up to 25–30% of people worldwide suffer from metabolic dysfunction-associated steatotic liver disease (MASLD), a metabolic condition characterized by an accumulation of fat in hepatocytes unrelated to alcohol consumption. The initial stages of MASLD are mostly asymptomatic and include simple steatosis (SS), of which 12–25% cases may progress to metabolic dysfunction-associated steatohepatitis (MASH) and which contributes to cirrhosis, liver cancer, and hepatic failure [1]. MASLD has been closely linked to obesity, type 2 diabetes, dyslipidemia, and hypertension, all of which together share components of a disturbed lipid and bile acid metabolism [2]. In addition, the changes observed in the activity of the gut microbiome, by acting on signaling via the gut–brain axis, have been implicated in mental health conditions [3], as well as in depression, which is considered a comorbid condition of MASLD. Interestingly, up to 67.5% of MASLD patients develop depressive symptoms [4], with the pooled prevalence being 40.68% in those with MASH [5]. Moreover, patients with psychiatric diseases seem to have a higher burden of metabolic disorders compared to the general population, and those with mental illness who are diagnosed with liver steatosis or fibrosis have worse metabolic profiles [6]. Given a potential bidirectional link between obesity and MASLD risk, along with the occurrence of depression, the role of gut dysbiosis—along with other disturbances, including oxidative stress and inflammation—can influence the pathogenesis of both conditions. For example, an altered metabolism of the neurotransmitters glutamine (Glu) and dopamine (DA) has been linked to abnormal hepatic function [7] characterized by elevated levels of the liver enzymes alanine aminotransferase (ALT), aspartate aminotransferase (AST), and gamma-glutamyl transferase (GGT) [7,8]. Also, altered bile acid (BA) homeostasis, reported in MASLD [9,10,11] and Major Depression Disorder (MDD) [3,12,13] patients, along with other metabolic disturbances, can increase inflammation and oxidative stress, potentially leading to an elevation of hepatic enzymes and liver damage [10].

BAs, steroid acids produced in the liver from cholesterol, can be classified into primary BAs, which are cholic acid and chenodeoxycholic acid derivatives conjugated with glycine or taurine to form bile salts, which are secreted into bile and stored in the gallbladder, and secondary BAs, such as deoxycholic acid and lithocholic acid, produced by gut bacteria from primary BAs. BAs acting as signaling metabolites through binding to specific receptors such as farnesoid X receptor (FXR) and G Protein-Coupled Bile Acid Receptor 1 (GPBAR1/TGR5) are involved in the regulation of metabolic homeostasis and also in neural signaling [14]. In addition, the presence of inflammation, oxidative stress, or gut dysbiosis, reported in both MASLD and MDD [4,5,7], can disrupt BAs synthesis and lead to changes in the bile acid pool, characterized by higher levels of ursodeoxycholic acid (UDCA), taurocholic acid (TCA), chenodeoxycholic acid (CDCA), taurochenodeoxycholic acid (TCDCA), and glycocholic acid (GCA) compared to healthy controls [9]. Also, impaired BA transport may contribute to the intrahepatic retention of hydrophobic BAs [10], such as TCA, taurodeoxycholic acid (TDCA), taurolithocholic acid (TLCA), and glycolithocholic acid (GLCA), which have been proposed as potential prognostic biomarkers of MASLD progression [9]. Liver dysfunction in MASLD can also impact the microbial fermentation and production of short-chain fatty acids (SCFAs), as patients with moderate to severe MASLD seem to exhibit lower levels of specific SCFAs, such as isobutyrate and methylbutyrate, compared to those in whom the condition is mild or those without the disease [15]. Notably, in obese individuals, the microbial production of SCFAs, especially butyrate, propionate, and acetate, has been reported to be dysregulated and has been proposed as a potential contributor to the development of low-grade systemic inflammation, insulin resistance, and impaired gut barrier function, which all together are key drivers of obesity-related complications, including mental health conditions [1,2,3] and brain diseases such as Alzheimer’s Disease [16]. Although depression has many risk factors, chronic stress, associated with the activation of the hypothalamic–pituitary–adrenal (HPA) axis [17], has been implicated in BA metabolism [18] and disease severity and treatment outcomes [19,20]. BA profiles differ significantly in patients with MDD compared to healthy controls, and changes in the levels of primary BAs (e.g., cholic acid, chenodeoxycholic acid) and secondary BAs (e.g., lithocholic acid, deoxycholic acid) are considered disruptors of signaling within the gut–brain axis and neurotransmitter metabolism [13,21], as well as within lipid digestion and glucose homeostasis, implicated in the development of neuro-inflammatory processes [1]. In MASLD, elevated BAs such as TLCA and GLCA have also been reported to be lower in MDD patients [18] when compared to controls, whereas gut-derived BAs produced from CDCA, including lithocholic acid (LCA), were significantly higher in more anxious individuals [13]. Similar changes were also found in SCFA profiles, as individuals with depressive symptoms tend to have reduced fecal concentrations of SCFAs [16], including acetic, propionic, and butyric acid, compared to controls [22]. This may be of particular importance, as alterations in the gut microbiome have been associated with changes in BA and SCFA profiles and have been implicated in the development of depressive symptoms [23] and also in metabolic conditions [24] such as type 2 diabetes [25], obesity [25], and MASLD [19,26].

Emerging evidence [27,28] supporting the role of microbiota-derived metabolites such as BAs and SCFAs in psycho-metabolic health suggests that targeting the gut–liver–brain axis may provide a promising avenue for developing new diagnostic and therapeutic strategies for MDD and obesity-associated conditions such as type 2 diabetes and MASLD. Therefore, this pilot study aims to investigate microbiota-derived metabolites, including BAs and SCFAs, in a cohort of women with morbid obesity at risk of MASLD and type 2 diabetes. This study will assess their relationship with depressive disorder (DD) and explore their potential as predictive biomarkers of DD. A secondary objective is to determine the levels of metabolic and inflammatory markers implicated in systemic and neuro-inflammation in these patients and its relationship with BAs and SCFAs.

## 2. Material and Methods

### 2.1. Study Population

This study was conducted in accordance with the ethical principles outlined in the Declaration of Helsinki. All participants provided informed consent prior to their inclusion in this study, and the protocol was approved by the institutional review board (Institut Investigació Sanitària Pere Virgili CEIm (Comité Ético de Investigación con medicamentos, Drug Research Ethics Committee in English): 23c/2015).

This pilot study was conducted on a cohort of 33 female participants with morbid obesity who underwent bariatric surgery at the Hospital Universitari Sant Joan de Reus between 2014 and 2017. We excluded men from this study in order to reduce variability an also since women represent the majority of bariatric surgery patients and show distinct metabolic and hormonal profiles.

The inclusion and exclusion criteria, biochemical analysis, and MASLD diagnoses were described previously [29]. Briefly, the patient was excluded from the study if she (1) had alcohol consumption higher than 10 g/d; (2) had acute or chronic hepatic, inflammatory, infectious, or neoplastic diseases; (3) had the status of menopausal woman or woman using contraceptives to avoid the interference of hormones that can cause biases in glucose and lipid metabolism; (4) had confirmed type 2 diabetes and received treatment with pioglitazone; and (5) had been treated with antibiotics in the previous 4 weeks. Patients who met the inclusion criteria were classified as patients with depressive disorder and control (CN) patients, based on whether they had been diagnosed with DD and/or were receiving antidepressant treatment (DD group, *n* = 10) or not (CN group, *n* = 23). It is important to note that both groups were patients with morbid obesity.

Hepatic samples were obtained during scheduled laparoscopic bariatric surgery in patients with suspected liver disease.

### 2.2. Hepatopathological Diagnosis

Liver samples were assessed by experienced hepatopathologists using the methods described elsewhere [30,31]. Briefly, liver samples collected during bariatric surgery were stained using hematoxylin–eosin and Masson’s trichrome stain. The degree of steatosis, inflammation, and ballooning and the presence of fibrosis in the patients were analyzed using the Kleiner criteria [30]. Based on these criteria, subjects were categorized into NL (normal liver) and MASLD patients; MASLD patients were further subdivided into two subgroups: SS (the initial stage of MASLD, characterized by steatosis without significant inflammation or ballooning) and MASH (defined by the presence of steatosis with inflammation and hepatocellular ballooning, with or without fibrosis).

### 2.3. Anthropometric Evaluation and Biochemical Analysis

The anthropometric measures and biochemical variables are described in Table 1.

Blood samples were extracted through a BD Vacutainer^®^ (BD IBERIA S.L., Madrid, Spain) system by trained hospital nurses immediately before surgery after overnight fasting. They were then separated into plasma and serum aliquots by centrifugation (1507 relative centrifugal force, 4 °C, 15 min) and stored at −80 °C for biochemical analysis of glucose, insulin, total cholesterol (TC), high-density lipoprotein cholesterol (HDL-C), low-density lipoprotein cholesterol (LDL-C), Glycated Hemoglobin A1c (HbA1c), and triglycerides, which was conducted on the automated analyzer Atellica Systems Analyser (Siemens Healthineers, Erlangen, Germany). Insulin resistance was determined using homeostatic model assessment 1 for insulin resistance (HOMA1-IR).

### 2.4. Plasma Measurements

The procedure of the measurement and quantification of microbial-derived metabolites, bile acids, metabolic biomarkers, and cytokines was based on [32] and was used to determine the serum levels of 11 BAs, including chenodeoxycholic acid (CDCA), deoxycholic acid (DCA), glycocholic acid (GCA), glycochenodeoxycholic acid (GCDCA), glycodeoxycholic acid (GDCA), taurochenodeoxycholic acid (TCDCA), taurodeoxycholic acid (TDCA), taurolithocholic acid (TLCA), tauroursodeoxycholic acid (TUDCA), glycolithocholic acid (GLCA), and glycoursodeoxycholic acid (GUDCA), by using liquid chromatography coupled to triple quadrupole mass spectrometry (LC-QqQ). In addition, other microbial-derived metabolites, such as choline, trimethylamine (TMA), trimethylamine N-oxide (TMAO), and betaine, along with SCFAs (acetic, butyric, and propionic acid) were also absolutely quantified by LC-QqQ. The circulating levels of cytokines implicated in systemic and neuroinflammation, such as interleukins (ILs) IL-1β, IL-6, IL-8, IL-10, IL-7, and TNF-α [33,34,35,36], were determined using multiplex sandwich immunoassays and a MILLIPLEX MAP Human High Sensitivity T Cell Magnetic Bead Panel (HSTCMAG-28SK-07, Millipore, Billerica, MA, USA), whereas adipocytokines linked to metabolic dysfunction [37,38], such as adiponectin, leptin, resistin, lipocalin, and Monocyte Chemoattractant Protein-1 (MCP-1), were measured using a MILLIPLEX MAP Human Adipokine Magnetic Bead Panel 1 (HADK1MAG-61K-04, Millipore, Billerica, MA, USA) and a MILLIPLEX MAP Human Adipokine Magnetic Bead Panel2 (HADK2MAG-61K-02, Millipore, Billerica, MA, USA). Both assays were analyzed on the Bio-Plex 200 instrument (Bio-Rad, Hercules, CA, USA), according to the manufacturer’s instructions.

### 2.5. Statistical Analysis

Data analyses were performed using a jupyter notebook (Python 3.11.8, Python Software Foundation, Wilmington, DE, USA). The distribution of variables was obtained using the Kolmogorov–Smirnov test. All results are expressed as medians and interquartile ranges (25th–75th). The different comparative analyses were performed using a nonparametric Mann–Whitney U test or using analysis of covariance (ANCOVA) when covariate adjustment was necessary. Where indicated, *p*-values were adjusted for multiple comparisons with the Benjamini–Hochberg method, and *p* values < 0.05 were considered statistically significant. The strength of association between variables was assessed with partial correlation, with type 2 diabetes as a covariate, using the pingouin package (version 0.5.4, Raphaël Vallat, Berkeley, CA, USA). Predictive models were built using the module scikit-learn (version 1.2.2, Inria Foundation, Paris, France [39]) using the LogisticRegression class with liblinear solver and balanced weight classes. In order to select the best features for the models, we used the SequentialFeatureSelector class (scikit-learn 1.2.2).

## 3. Results

### 3.1. Characteristics of Study Participants

In this pilot study, a total group of 33 women with morbid obesity were included. In this cohort, 30.3% had a DD diagnosis and/or received antidepressants (DD group, *n* = 10), while others reported no depression symptoms nor antidepressant treatment (control group: CN, *n* = 23). The calculated DD prevalence in this cohort was 30.3%. The clinical characteristics and biochemical parameters of the cohort are presented in Table 1.

All participants were compared in terms of general anthropometric parameters, including height, BMI, and systolic (SBP) and diastolic (DBP) blood pressure, as well as indicators of metabolic status (e.g., HOMA index, glycosylated hemoglobin (HbA1c), and glucose and insulin blood levels), lipid profile (e.g., cholesterol, high-density lipoprotein cholesterol (HDL-C), low-density lipoprotein cholesterol (LDL-C), and triglycerides), and indicators of liver metabolism (e.g., AST, ALT, GGT, and alkaline phosphatase (ALP)) and inflammation (C-reactive protein (CRP)) (Table 1). There were no significant differences between the groups in terms of anthropometric and biochemical measures.

Patients in this cohort were affected by morbid obesity in addition to other metabolic conditions; therefore, they were under ongoing treatment with medications necessary to manage chronic symptoms. The results indicated that females in the DD group had a significantly higher intake of medications related to type 2 diabetes treatment and received insulin, and they had a significantly higher intake of pain medications, including analgesics (*p* < 0.05) (Table 2).

The women in this cohort were also affected by other comorbidities associated with obesity, including type 2 diabetes (*n* = 13, 39.4% of total cohort), high blood pressure (*n* = 19, 57.6% of total cohort), and MASLD (*n* = 22, 66.7% of total cohort), including steatosis (*n* = 22, 66.7% of total cohort) and steatohepatitis (*n* = 8, 24.2% of total cohort). The comparisons of the co-incidence of comorbid metabolic conditions between the DD group and CN group are presented in Table 3. The women classified into the DD group had a significantly higher incidence of type 2 diabetes compared to the CN group (*p* = 0.018). Nevertheless, there were no significant differences in the incidence of MASLD, metabolic syndrome, and high blood pressure between the groups.

### 3.2. Evaluation of Microbiota-Derived Metabolites Between the Study Groups

**Figure 1:**
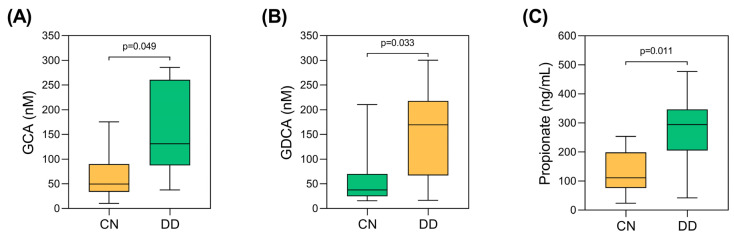
Significant changes in levels of microbiota-derived metabolites (**A**) GCA, (**B**) GDCA, and (**C**) propionate between control group (CN) and depression disorder cohort (DD). *p* values were calculated by using ANCOVA, adjusting for type 2 diabetes diagnosis, and by multiple comparisons with Benjamini–Hochberg method. *p* < 0.05 was considered statistically significant.

The first objective of this study was to determine the levels of microbiota-derived metabolites, as BAs and SCFAs, in blood samples in relation to the presence of DD. There was a marked difference in the proportion of patients with diabetes between the CN and DD groups, with 26% of the CN group and 70% of the DD group diagnosed with type 2 diabetes. Therefore, following adjustment for type 2 diabetes diagnosis (Figure 1; Table S1, Supplementary Material), the results showed that in the DD group, the levels of BAs, GCA (Figure 1A), and GDCA (Figure 1B) were significantly higher compared to the CN group (*p* < 0.05). In addition, SCFAs, such as propionate (Figure 1C), were significantly elevated in those with DD compared to those without DD (*p* < 0.05). There were no statistical differences between the groups in terms of the concentrations of other microbial-derived bioactives (*p* < 0.05).

### 3.3. Evaluation of Metabolic and Inflammatory Biomarkers in the Cohort

A secondary objective of this study was to determine the levels of metabolic and inflammatory markers implicated in inflammation, such as IL-1β, IL-6, IL-8, IL-10, IL-7, and TNF-α [33,34,35,36,37,38], along with markers of metabolic dysfunction, including adiponectin, leptin, resistin, lipocalin, and MCP-1 [37,38], in the blood samples collected from the participants in the presence of DD (Table S2, Supplementary Material). The results indicated that women in the DD group had significantly higher levels of IL-1β and leptin compared to the CN group (*p* < 0.05); however, this significance was lost upon adjustment to type 2 diabetes diagnosis.

### 3.4. Correlations Between Microbiota-Derived Metabolites and Measured Biomarkers

To assess the relationship between microbiota-derived metabolites and the measured immuno-metabolic biomarkers, a series of correlations with Spearman’s ratio was conducted, and it is presented in the subsections below.

#### 3.4.1. Correlations Between BAs and SCFAs and Other Microbial Bioactives

The correlations between BAs, SCFAs and other microbiota bioactives are presented in Figure 1. The results indicated significant positive relationships between certain secondary BAs and SCFAs, with the exception of acetate, which was negatively correlated with CDCA. Also, the other microbial metabolites, namely TMA, TMAO, and choline, were positively associated with certain BAs, such as DCA and TDCA (Figure S1, Supplementary Material).

#### 3.4.2. Correlations Between Microbiota Metabolites and Clinical Characteristics

**Figure 2:**
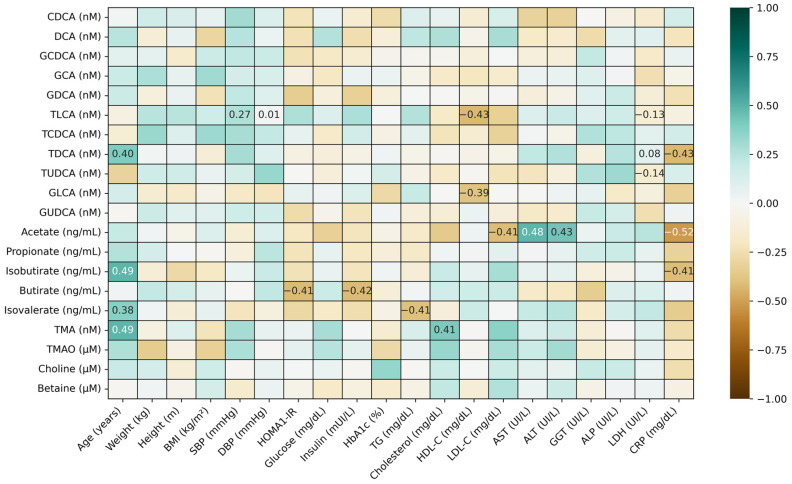
Partial correlations between bile acids (BAs), short-chain fatty acids (SCFAs), and other microbial bioactives and the clinical and biochemical characteristics of the study cohort (using diabetes mellitus as a covariate). Spearman’s rank coefficient is displayed only in the cases of significant (*p* value < 0.05) associations. CDCA, Chenodeoxycholic acid; DCA, Deoxycholic acid; GCDCA, Glycochenodeoxycholic acid; GCA, Glycocholic acid; GDCA, Glycodeoxycholic acid; TCLA, Taurochenodeoxycholic acid; TCDCA, Taurochenodeoxycholic acid; TDCA, Taurodeoxycholic acid; TUDCA, Tauroursodeoxycholic acid; GLCA, Glycolithocholic acid; GUDCA, Glycoursodeoxycholic acid; TMA, Trimethylamine; TMAO, Trimethylamine N-oxide; BMI, body mass index; SBP, systolic blood pressure; DBP, diastolic blood pressure; HOMA1-IR, homeostatic model assessment method–insulin resistance; HbA1c, glycosylated hemoglobin; TG, triglycerides; HDL-C, high-density lipoprotein cholesterol; LDL-C, low-density lipoprotein cholesterol; AST, aspartate aminotransferase; ALT, alanine aminotransferase; GGT, gamma-glutamyltransferase; ALP, alkaline phosphatase; LDH, Lactate Dehydrogenase; CRP, C-reactive protein. All BAs are measured as nM; SCFAs are measured as ng/mL;TMA determined as nM. Choline, TMAO, and betaine are measured as uM.

The correlations between microbial metabolites and the measured clinical characteristics of the study cohort are presented in Figure 2. The analysis showed significant positive correlations (*p* < 0.05) between BAs, namely TDCA, isobutyrate, isovalerate, and TMA, and age, whereas SBP and DBP were positively correlated with another BA, namely TLCA. The liver function biomarkers AST and ALT were positively correlated with acetate levels, whereas the inflammatory marker CRP was negatively correlated with this SCFA. A series of negative correlations was also observed between HOMA-1R, insulin, and triglyceride levels and two SCFAs, namely butyrate and isovalerate (*p* < 0.05) (Figure 2).

#### 3.4.3. Correlations Between Microbial Metabolites and Immuno-Metabolic Biomarkers

**Figure 3:**
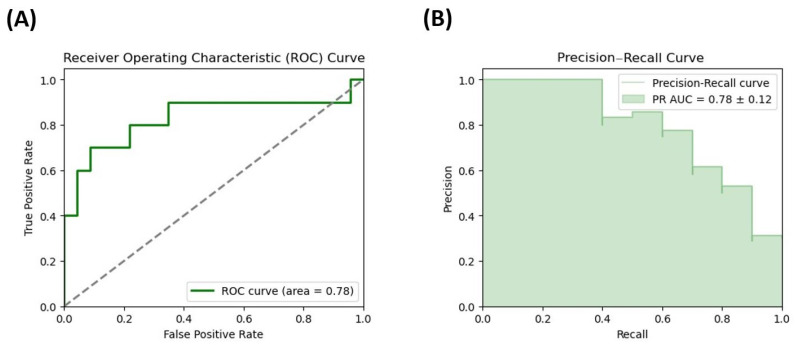
ROC curve and precision–recall curve for microbial metabolites. (**A**) ROC curve for glycodeoxycholic acid (GDCA), the bile acid (BAs) selected by the best-fit method, in a logistic regression model predicting depression disorder (DD) in a cohort of females with morbid obesity. (**B**) Precision–recall curve for glycodeoxycholic acid (GDCA), the bile acid (BAs) selected by the best-fit method, in a logistic regression model predicting depression disorder (DD) in a cohort of females with morbid obesity. The dashed diagonal line in the ROC plot represents the performance of a random classifier (AUC = 0.5).

The correlation analysis between microbial metabolites and the measured immuno-metabolic biomarkers is presented in Figure 3. In this cohort, most of the BAs, SCFAs, and other microbial bioactives were negatively correlated with immune and metabolic measures (*p* < 0.05), with the exception of leptin, the levels of which were positively correlated with BAs, including GCDCA, GCA, TLCA, TDCA, and TUDCA (*p* < 0.05) (Figure S2).

### 3.5. Evaluation of the Potential of Microbiota Metabolites as Potential Biomarkers of DD Risk in Female Patients with Severe Obesity

**Figure 4:**
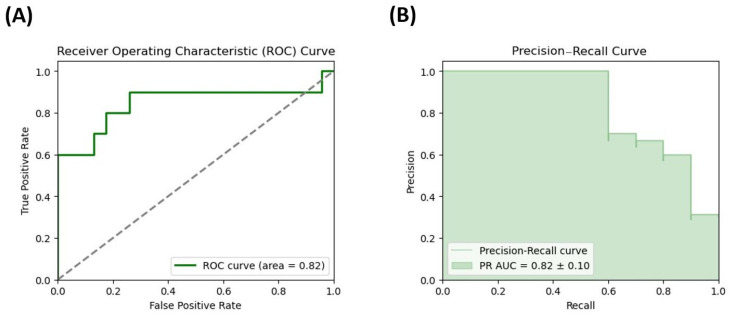
ROC curve and precision–recall curve for propionate. (**A**) ROC curve for propionate, the predictor selected by the best-fit method among all measured variables in a logistic regression model predicting depression disorder (DD) in a cohort of females with morbid obesity. (**B**) Precision–recall curve for propionate, the predictor selected by the best-fit method among all measured variables in a logistic regression model predicting depression disorder (DD) in a cohort of females with morbid obesity. The dashed diagonal line in the ROC plot represents the performance of a random classifier (AUC = 0.5).

To determine the potential of the measured microbiota metabolites in predicting the occurrence of DD symptoms in the women in our cohort, the receiver operating characteristic (ROC) curve and the precision–recall curve of the logistic regression model were obtained for two microbiota metabolites selected by the best fit-method, GDCA and propionate. The Area Under the Curve (AUC) for the model including GDCA was 0.780 and the odds ratio value was 5.12 (Figure 3), whereas the prediction accuracy was 82% and the sensitivity was 87%. Furthermore, logistic regression analyses helped to reveal the highest AUC value of 0.82 for propionate, with an obtained odds ratio of 5.15 (Figure 4), and model performance demonstrated an accuracy of 79%, a sensitivity of 94%, and a specificity of 60%.

## 4. Discussion

There is growing evidence [27,28] suggesting the presence of a bidirectional link between the gut microbiome, including microbial-derived metabolites such as BAs and SCFAs, and brain and liver health. Consequently, the exploration of the gut–bile acid axis may offer novel strategies for the management of psychiatric symptoms in patients with high metabolic risks. Therefore, the key objective of this early exploratory study was to analyze microbiota-derived metabolites, specifically BAs and SCFAs, in a cohort of females with morbid obesity at high risk of MASLD and type 2 diabetes in relation to DD status and to assess the further potential of these metabolites as potential predictors of DD. A secondary objective was to determine the levels of metabolic and inflammatory markers implicated in metabolic health and DD symptoms and their relationship with BAs and SCFAs.

The main results showed that in this cohort of women with comorbid obesity, the prevalence of DD symptoms was equal to 30%, and these females presented significantly higher levels of certain microbiota-derived metabolites, including GCA, GDCA, and propionate. Among these, GDCA and propionate had the best performance in predicting DD as evaluated by ROC analysis, with accuracies of 82% and 79%, respectively. Furthermore, certain BAs, SCFAs, and other microbiota-derived metabolites were significantly correlated with age, SBP, DBP, insulin resistance/insulin levels, lipid profile, and liver and inflammatory biomarkers, as well as inflammatory and metabolic indices, such as IL-1β and leptin. These correlations may be relevant to the health status of participants, as some females, particularly those with DD symptoms, reported other metabolic complications, as well as higher intakes of anti-diabetic and analgesic medications.

The prevalence of DD in this cohort was consistent with other studies indicating that between 33% and 40% of females experience depressive symptoms prior to undergoing bariatric surgery [30,40]. In addition, females in the DD group had a significantly higher presence of type 2 diabetes compared to those without DD symptoms and had a significantly higher intake of anti-diabetic and pain medications, including insulin and analgesics. This is consistent with other studies, which have shown that women with depression are more likely to use antidepressants and pain medications [41] to mitigate chronic pain and also receive anti-diabetic treatments to manage their glycemic status [42,43]. However, in the present pilot study, we were not able to find differences in the incidence of MASLD or metabolic syndrome between patients with or without DD. These inconsistencies seem to be prevalent in other studies, which, despite showing independent relationship between newly formed MAFLD and depression, failed to demonstrate an associations between depression score and clinically significant liver fibrosis [44], as well as progression to major adverse liver outcomes such as decompensated cirrhosis, hepatocellular carcinoma, or liver transplantation [45].

Regarding microbiota-derived metabolites, females with depressive disorder had a different profile of BAs, SCFAs, and other microbiota-derived bioactives compared to those without DD, which may suggest the presence of certain changes in the gut microbiome of individuals with DD [46]. Moreover, the levels of propionate were significantly higher in females in the DD group compared to controls, and these levels were significantly correlated with GCA and GDCA. Although associations between SCFAs and BAs were reported in MDD and also in MASLD [20,47], this finding should be interpreted with caution, as the levels of SCFAs have been shown to vary significantly [3,9,11,16,22] in these cohorts compared with controls. One study noted that increased levels of isovalerate were associated with higher cortisol levels in patients [48], while those with mild or moderate MASLD seem to have increased levels of butyrate and propionate [49]. In the present study, isobutyrate was correlated with age and the inflammatory marker CRP, as well as some cytokines, such as IL-6 and IL-8, whereas isovalerate was correlated with age, triglycerides, resistin, and inflammatory cytokines, including IL-6, IL-1β, TNF-a, and IL-7. Propionate was associated with inflammatory biomarkers such as IL-1 β and TNF-a. The specific roles of BAs and SCFAs in psycho-metabolic health remain unclear. However, early research suggests they may play a role in signaling within the gut–liver–brain axis [50,51]. Secondary BAs, as agonists of FXR and TGR5, could also influence lipid and carbohydrate metabolism. For example, TGR5 activation has been associated with an increased expression of mitochondrial genes and inflammatory pathways upon neuroinflammation [50] and with the formation of >beiging in subcutaneous white adipose tissue and lipolysis in experimental models [51]. Similarly, SCFAs, as endogenous ligands for G protein-coupled receptors (GPCRs), particularly GPR41 (FFAR3) and GPR43 (FFAR2) [52], can influence the production of gut hormones implicated in regulation of appetite, glycemic and energy homeostasis, and immune and neural signaling [16]. Moreover, propionate, by modulating the expression of phosphoenolpyruvate carboxykinase (PEPCK) and glucose-6-phosphatase (G6Pase), has been implicated in glucose homeostasis and in regulating gluconeogenesis in the liver [50]. It is noteworthy that, by influencing the expression of protein-coupled receptors (GPCRs) such as GPR41 and GPR43 via direct action on Histone Deacetylase (HDAC) [53], SCFAs can modulate immune homeostasis, which in the case of psycho-metabolic conditions is skewed towards pro-inflammatory responses with increased levels of CRP, IL-1β, IL-6, and TNF-α [54,55]. These observations are consistent with previous studies [9,10] investigating BAs and SCFAs as predictors of MASLD [9] and MDD [18,23,56]. Our exploratory analyses revealed a positive correlation between GDCA and GCA levels and leptin, suggesting that certain BAs may play a role in metabolic changes. However, further research is needed to confirm and clarify this potential relationship.

Given the exploratory nature of this pilot study, our findings should be interpreted with caution due to several limitations, including a small sample size, a focus on women, and the absence of dietary habit assessments and formal psychiatric diagnoses. As classification into the DD group was based on clinical history reported by a doctor, this analysis lacked validated data, which may influence diagnostic rigor. It is noteworthy that, due to the transversal nature of this research, the individuals recruited in our cohort had BAs, SCFAs, and other microbiota-derived bioactives determined only before their bariatric surgery; therefore, we cannot establish whether the changes in microbiota-related metabolites reported in this study are the cause and/or consequence of the procedure. Also, participants were prescribed medications, including antidepressants, anti-diabetic medications, and analgesics, which may impact the physiology of the gut microbiome, brain, and liver. Therefore, to confirm these results, more research with a comprehensive psychiatric and dietary assessment, as well as a bigger sample with a wide range of backgrounds representing both sexes, is needed. For further studies, it would also be beneficial to determine the profile of BAs, SCFAs, and other microbiota-derived bioactives not only before bariatric surgery, but also after the medical procedure, as a follow-up, to assess potential changes caused by the intervention.

## 5. Conclusions

To conclude, this exploratory study, conducted with a small cohort of females with morbid obesity undergoing bariatric surgery and reporting depressive disorder (DD) symptoms, suggests a distinct gut microbial metabolite profile associated with DD. This profile is characterized by significantly elevated levels of microbial-derived bile acids (GCA, GDCA) and short-chain fatty acids (propionate). Moreover, our preliminary results highlight the potential value of assessing immuno-metabolic changes in females experiencing both depressive symptoms and metabolic conditions. Nevertheless, further research is needed to explore this observation as a novel avenue for biomarker discovery.

## Figures and Tables

**Table 1:** Anthropometric and biochemical variables in the studied cohort.

Variables	Total Cohort (*n* = 33)	Depression Disorder (DD) Group (*n* = 10)	Control (CN) Group (*n* = 23)	*p* Value
Age (years)	49.39 (41.39–57.53)	51.48 (42.43–56.98)	48.54 (37.12–54.40)	0.232
Weight (kg)	116.00 (110.10–130.50)	118.00 (110.75–128.50)	116.00 (111.10–132.00)	0.953
Height (m)	1.60 (1.58–1.67)	1.58 (1.56–1.60)	1.62 (1.58–1.68)	0.146
BMI (kg/m^2^)	44.06 (42.35–48.73)	44.10 (43.74–50.23)	43.93 (42.36–47.86)	0.518
SBP (mmHg)	132.00 (107.50–138.50)	130.50 (107.25–138.00)	132.00 (109.75–138.00)	0.951
DBP (mmHg)	63.00 (57.75–74.75)	61.50 (59.25–73.50)	65.00 (57.75–72.50)	0.976
HOMA1-IR	3.60 (1.77–7.34)	6.82 (3.32–17.40)	2.84 (1.58–4.71)	0.060
Glucose (mg/dL)	107.00 (87.00–148.00)	138.00 (110.75–157.50)	100.00 (86.50–112.50)	0.088
Insulin (mUI/L)	13.64 (6.92–31.19)	22.60 (14.18–48.76)	12.18 (6.35–17.95)	0.150
HbA1c (%)	5.75 (5.38–7.83)	7.55 (5.68–8.12)	5.60 (5.30–6.15)	0.078
TG (mg/dL)	132.00 (105.00–166.00)	134.00 (121.50–152.50)	132.00 (103.00–187.00)	0.949
Cholesterol (mg/dL)	169.00 (153.00–188.00)	165.00 (133.57–189.25)	170.00 (154.00–186.20)	0.597
HDL-C (mg/dL)	37.40 (30.88–46.25)	36.00 (32.00–48.00)	37.80 (31.00–44.00)	0.839
LDL-C (mg/dL)	96.25 (79.15–120.05)	90.20 (66.10–114.20)	100.00 (92.60–118.60)	0.189
AST (UI/L)	28.50(19.00–45.00)	22.00 (15.00–36.00)	32.00 (22.00–45.00)	0.341
ALT (UI/L)	33.00 (22.00–45.00)	26.50 (20.25–43.00)	34.00 (24.00–43.00)	0.512
GGT (UI/L)	21.00 (16.50–32.50)	29.00 (18.25–34.75)	18.00 (15.50–26.50)	0.098
ALP (Ul/L)	69.00 (53.50–79.25)	72.00 (53.00–74.00)	64.00 (56.50–77.50)	0.941
LDH (Ul/L)	405.50 (354.75–457.25)	330.00 (296.50–419.50)	406.00 (374.50–457.50)	0.185
CRP (mg/dL)	0.70 (0.45–1.60)	0.60 (0.40–1.30)	0.70 (0.50–1.50)	0.686

BMI, body mass index; SBP, systolic blood pressure; DBP, diastolic blood pressure; HOMA1-IR, homeostatic model assessment method–insulin resistance; HbA1c, glycosylated hemoglobin; TG, triglycerides; HDL-C, high-density lipoprotein cholesterol; LDL-C, low-density lipoprotein cholesterol; AST, aspartate aminotransferase; ALT, alanine aminotransferase; GGT, gamma-glutamyltransferase; ALP, alkaline phosphatase; LDH, Lactate Dehydrogenase; CRP, C-reactive protein. Data are expressed as medians (interquartile ranges), except sex, which is displayed as the percentage (%) of females. The independent-samples comparison was made with Mann–Whitney U tests.

**Table 2:** The reported medication intake (*n*, %) in the studied cohort in respect to study groups.

Medications/Treatment	Total Cohort (*n* = 33)	Depression Disorder (DD) Group (*n* = 10)	Control (CN) Group (*n* = 23)	*p* Value
Antihypertensive	19 (57.6%)	8 (24.2%)	11 (33.3%)	0.089
Lipid-lowering agents—statins	8 (24.2%)	4 (12.1%)	4 (12.1%)	0.164
Lipid-lowering agents—fibrates	2 (6.1%)	1 (3.05%)	1 (3.05%)	0.532
Type 2 diabetes treatment—insulin *	7 (21.2%)	5 (15.2%)	2 (6.1%)	0.008
Type 2 diabetes treatment—oral *	13 (39.4%)	7 (21.2%)	6 (18.2%)	0.018
Analgesics	3 (9.1%)	2 (6.1%)	1 (3.0%)	0.151
Opioid analgesics *	2 (6.1%)	2 (6.1%)	0 (0%)	0.027
Antibiotics	0 (0%)	0 (0%)	0 (0%)	-
Anti-inflammatory drugs	6 (18.2%)	3 (9.1%)	3 (9.1%)	0.246
Benzodiazepines	3 (9.1%)	2 (6.1%)	1 (3.0%)	0.151
Cytostatic immunosuppressants	1 (3.0%)	1 (3.0%)	0 (0%)	0.124
Corticosteroids	2 (6.1%)	1 (3.0%)	1 (3.0%)	0.532

Data are expressed as the number of participants (%) in the total cohort. Comparisons between groups were made by cross-tabs with the chi-square test. * Significant differences between the DD and CN group (*p* < 0.05).

**Table 3:** The reported differences in the incidence of comorbid conditions in our bariatric patient cohort in relation to depression diagnosis.

Comorbidity		Depression Disorder (DD) Group (*n* = 10)	Control (CN) Group (*n* = 23)	Xi^2^ (df)	*p* Value
T2DM *	No	3	17	5.629 (1)	0.018
	Yes	7	6		
Metabolic Syndrome	No	4	5	1.172 (1)	0.279
	Yes	6	18		
High Blood Pressure	No	2	12	2.954 (1)	0.086
	Yes	8	11		
MASLD	No	3	8	0.072 (1)	0.789
	Yes	7	15		

df, degree of freedom; T2DM, type 2 diabetes mellitus; MASLD, metabolic dysfunction-associated steatotic liver disease. Differences between the groups are determined by the chi-square test. * *p* < 0.05 was considered statistically significant.

## Data Availability

The data supporting the findings of this study are available within the publication and upon reasonable request to the corresponding author.

## Supplementary Materials

The following supporting information can be downloaded at https://www.mdpi.com/article/10.3390/cimb47050353/s1.
