# Supplementary material for: Differential Profiles of Gut Microbiota-Derived Metabolites of Bile Acids and Propionate as Potential Predictors of Depressive Disorder in Women with Morbid Obesity at High Risk of Metabolic Dysfunction-Associated Steatotic Liver Disease—A Pilot Study
Source: Curr Issues Mol Biol. 2025 May 12;47(5):353. doi: 10.3390/cimb47050353 (PMC12110740; doi:10.3390/cimb47050353)
Supplement: Supplementary file 1 [file cimb-47-00353-s001.zip › cimb-3606786-supplementary.pdf]

current issues in molecular biology

Supplementary Material

Table S1. Reported concentration of microbial metabolites, including bile acids and SCFAs in the blood samples in the studied cohort in respect to study groups.

Microbial metabolites	Total cohort (*n* = 33)	Depression Disorder (DD) group (*n* = 10)	Control (CN) group (*n* = 23)	Adjusted P value
**Microbial metabolites**				
CDCA (10;23)	29.91 (12.96-120.49)	59.20 (20.99-137.44)	29.91 (11.92-98.21)	0.491
DCA (10;23)	139.80 (52.05-296.80)	293.56 (175.23-470.21)	95.71 (43.98-178.96)	0.413
GCDCA (10;23)	153.10 (69.47-321.30)	334.08 (158.65-405.09)	115.59 (54.09-197.22)	0.187
**GCA (10;22)***	**72.73 (35.56-137.40)**	**131.14 (93.96-234.40)**	**49.37 (34.23-88.98)**	**0.049**
**GDCA (10;23)***	**54.55 (26.77-147.40)**	**169.45 (85.87-214.88)**	**37.53 (25.16-66.80)**	**0.033**
TCLA (10;17)	1.22 (0.82-2.16)	1.48 (1.20-2.24)	0.95 (0.81-1.48)	0.491
TCDCA (10;23)	41.84 (20.24-98.20)	28.90 (25.76-107.50)	43.41 (20.24-78.12)	0.491
TDCA (9;22)	9.41 (4.85-32.88)	32.88 (12.18-36.13)	6.88 (3.55-12.50)	0.187
TUDCA (9;22)	3.06 (1.55-4.62)	4.21 (2.23-5.50)	2.16 (1.47-4.36)	0.239
GLCA (10;23)	25.66 (13.09-38.64)	43.42 (33.09-100.16)	15.02 (11.87-25.80)	0.980
GUDCA (10;23)	341.92 (70.07-780.10)	781.68 (407.89-1108.03)	188.56 (70.06-414.14)	0.187
**Short Chain Fatty Acids**				
Acetate (10;23)	2013.90 (1034.40-2689.90)	1969.60 (1129.99-2168.62)	2057.65 (1035.32-2689.98)	0.389
**Propionate (10;23)***	**119.80 (85.25-242.70)**	**294.50 (211.48-336.14)**	**111.14 (80.22-171.37)**	**0.011**
Isobutyrate (10;23)	27.56 (23.18-32.53)	32.14 (28.51-36.52)	25.25 (22.42-30.02)	0.187
Butyrate (10;23)	55.06 (38.56-75.29)	63.39 (39.40-66.73)	53.13 (38.94-75.28)	0.550
Isovalerate (10;23)	11.57 (8.02-19.83)	17.95 (10.63-27.26)	10.13 (7.56-13.64)	0.187
**Other microbial bioactives**				
TMA (10;23)	35.01 (25.67-55.05)	55.33 (33.51-83.15)	34.21 (23.82-43.63)	0.187
TMAO (10;23)	2.28 (1.66-3.68)	2.74 (2.07-5.86)	2.21 (1.40-2.92)	0.819
Choline (10;23)	20.47 (15.54-26.94)	26.10 (21.12-27.78)	19.03 (14.19-23.67)	0.413
Betaine (10;23)	25.29 (21.48-35.12)	36.43 (32.93-39.92)	23.96 (21.08-28.65)	0.187

CDCA, Chenodeoxycholic acid; DCA, Deoxycholic acid; GCDCA, Glycochenodeoxycholic acid; GCA, Glycocholic acid; GDCA, Glycodeoxycholic acid; TCLA, Taurochenodeoxycholic acid; TCDCA, Taurochenodeoxycholic acid; TDCA, Taurodeoxycholic acid; TUDCA, Tauroursodeoxycholic acid; GLCA, Glycolithocholic acid; GUDCA, Glycoursodeoxycholic acid; TMA, Trimethylamine; TMAO, Trimethylamine N-oxide; SCFAs, Short Chain Fatty Acids. All BAs are measured as nM; SCFAs are measured as ng/mL; TMA is determined as nM. Choline, TMAO, Betaine measured as uM. Data are expressed as the median (interquartile range). The independent sample comparison was made with Mann-Whitney U tests.

P values referring to the coefficient for the group (DD or CN) were calculated by using ANCOVA, adjusting for type 2 diabetes diagnosis; these were later adjusted for multiple comparisons with Benjamini-Hochberg method.

* Significant differences between DD and CN group (adjusted $P < 0.05$) presented in bold.

Table S2. Reported concentration of metabolic and inflammatory biomarkers in the studied cohort regarding to study groups.

Biomarker	Total cohort (*n* = 33)	Depression Disorder (DD) group (*n* = 10)	Control (CN) group (*n* = 23)	Adjusted P value
IL-1β (10;23)	4.08 (2.86-6.66)	3.16 (2.52-3.46)	5.48 (3.71-7.93)	0.115
IL-6 (10;22)	3.57 (2.39-6.21)	3.00 (1.58-4.16)	4.94 (2.75-7.13)	0.115
IL-7 (9;23)	6.75 (4.383-12.50)	4.97 (4.04-6.40)	8.25 (5.68-13.44)	0.115
IL-8 (10;22)	3.54 (2.49-4.345)	2.90 (2.28-4.30)	3.58 (2.68-4.12)	0.212

TNF-alpha (10;23)	10.77 (7.52-14.95)	9.46 (7.26-11.19)	10.82 (8.51-15.40)	0.212
Resistin (10;23)	32.84 (23.49-38.77)	26.16 (18.56-32.41)	34.94 (29.35-46.69)	0.115
Lipocalin-2 (10;23)	124.50 (94.39-148.30)	110.21 (90.06-139.98)	126.74 (101.88-154.70)	0.212
Adiponectin (10;23)	11560.40 (7319.80-18302.90)	7753.08 (5123.22-8349.05)	14262.14 (9584.42-18302.91)	0.131
Leptin (9;12)*	21584.70 (15239.40-29176.30)	27873.95 (20212.30-38624.27)	20068.94 (14670.92-24015.05)	0.115
MCP-1 (9;12)	97.69 (71.01-113.80)	75.82 (66.79-93.22)	105.42 (97.65-116.82)	0.115

IL, interleukin; TNF, tumour necrosis factor; GIP, glucose-dependent insulinotropic polypeptide; MCP-1, Monocyte Chemoattractant Protein-1. All Inflammatory biomarkers are measured in pg/mL; whereas metabolic biomarkers are displayed in ng/mL, with exception for ghrelin, GIP, PYY, leptin and MCP-1 which are presented as pg/mL. Due to limited sample material, the exact numbers of samples measured are displayed along with each microbial metabolite as (DD; CN). Data are expressed as the median (interquartile range). The independent sample comparison was made with Mann-Whitney U tests. *P* values are adjusted for multiple comparisons with Benjamini-Hochberg method.
* Significant differences between DD and CN group (*P* < 0.05).

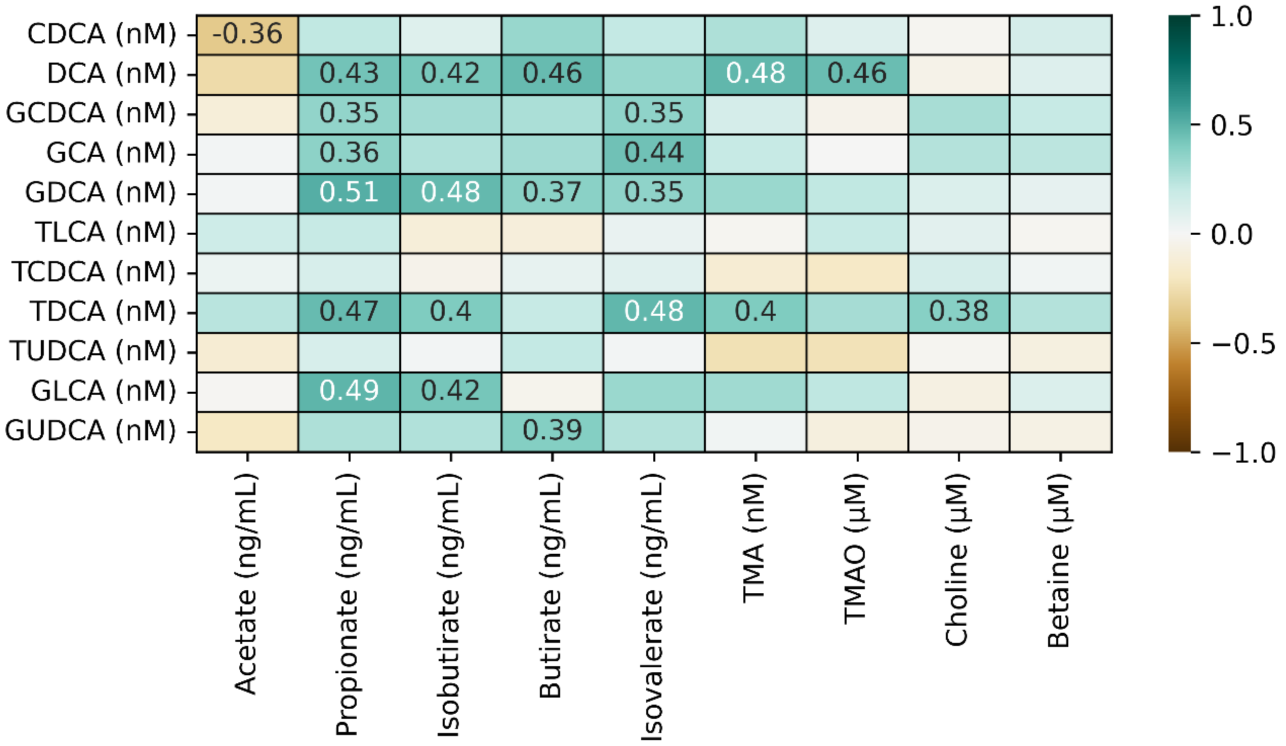

Figure S1. Partial correlations between bile acids (BAs) and short chain fatty acids (SCFAs) and other microbial bioactives (using diabetes mellitus as a covariate). Spearman's rank coefficient is displayed only in the cases of significant (P value < 0.05) associations. CDCA, Chenodeoxycholic acid; DCA, Deoxycholic acid; GCDCA, Glycochenodeoxycholic acid; GCA, Glycocholic acid; GDCA, Glycodeoxycholic acid; TCLA, Taurochenodeoxycholic acid; TCDCA, Taurochenodeoxycholic acid; TDCA, Taurodeoxycholic acid; TUDCA, Tauroursodeoxycholic acid; GLCA, Glycolithocholic acid; GUDCA, Glycoursodeoxycholic acid; TMA, Trimethylamine; TMAO, Trimethylamine N-oxide; SCFAs, Short Chain Fatty Acids. All BAs are measured as nM; SCFAs are measured as ng/mL; TMA determined as nM. Choline, TMAO, Betaine measured as uM.

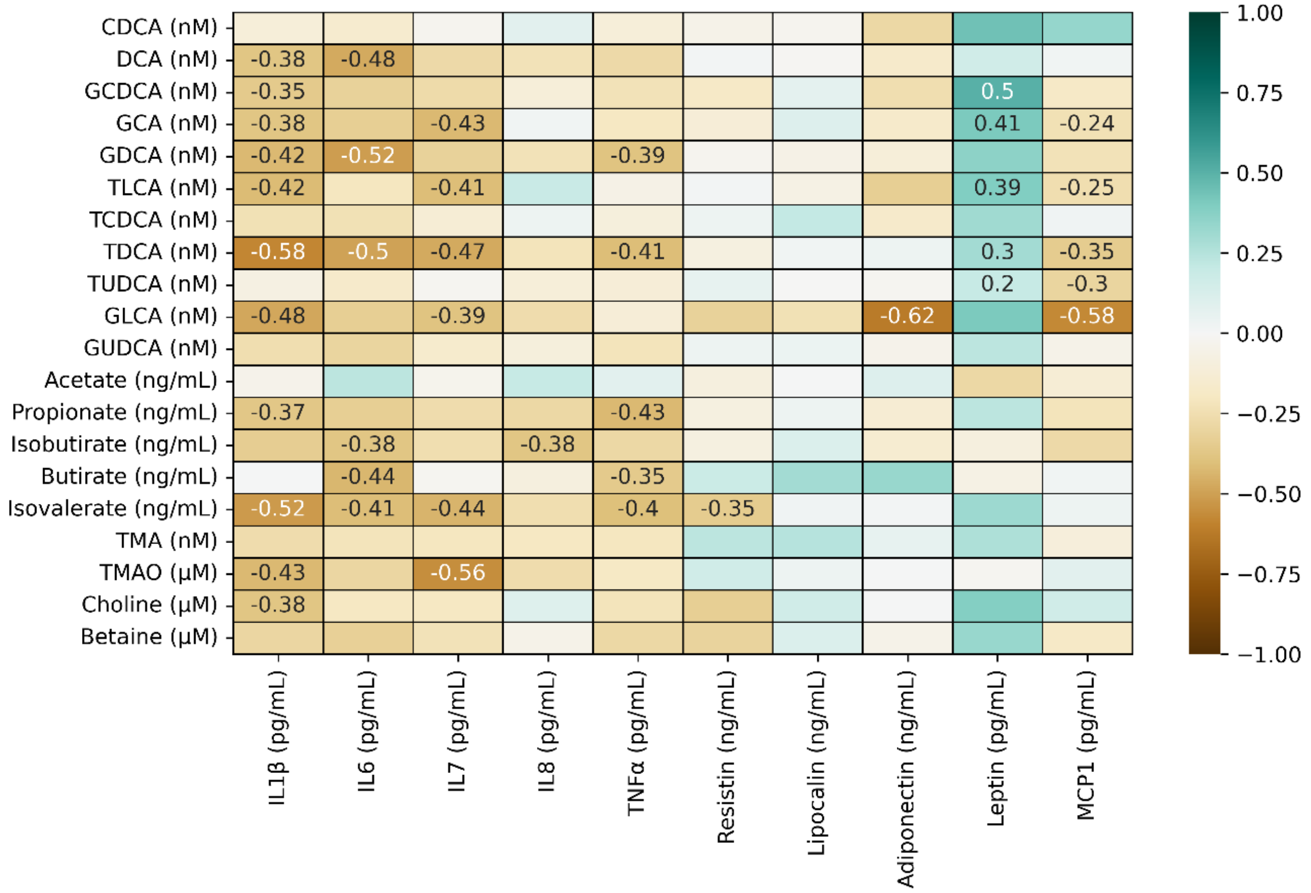

Figure S2. Partial correlations between bile acids (BAs) and short chain fatty acids (SCFAs) and other microbial bioactives with measured immune-metabolic markers of the study cohort (diabetes mellitus used as a covariate). Spearman's rank coefficient is displayed only in the cases of significant (P value < 0.05) associations.

CDCA, Chenodeoxycholic acid; DCA, Deoxycholic acid; GCDCA, Glycochenodeoxycholic acid; GCA, Glycocholic acid; GDCA, Glycodeoxycholic acid; TCLA, Taurochenodeoxycholic acid; TCDCA, Taurochenodeoxycholic acid; TDCA, Taurodeoxycholic acid; TUDCA, Tauroursodeoxycholic acid; GLCA, Glycolithocholic acid; GUDCA, Glycoursodeoxycholic acid; TMA, Trimethylamine; TMAO, Trimethylamine N-oxide; SCFAs, IL, interleukin; TNF, tumour necrosis factor; GIP, glucose-dependent insulinotropic polypeptide; MCP-1, Monocyte Chemoattractant Protein-1. All BAs are measured as nM; SCFAs are measured as ng/mL;TMA determined as nM. Choline, TMAO, Betaine measured as uM.
